# Genotype, Vernalization Duration and Nutrition Interactions in Sugar Beet Speed Breeding

**DOI:** 10.3390/plants15121850

**Published:** 2026-06-15

**Authors:** Aleksandra Yu. Kroupina, Pavel Yu. Kroupin, Mariya N. Polyakova, Malak Alkubesi, Alana A. Ulyanova, Daniil S. Ulyanov, Natalya Yu. Svistunova, Victoria Yu. Kanunnikova, Sergey Yu. Shirnin, Alina A. Kocheshkova, Gennady I. Karlov, Mikhail G. Divashuk

**Affiliations:** All-Russian Research Institute of Agricultural Biotechnology, Moscow 127434, Russia

**Keywords:** *Beta vulgaris* L. var. *altissima*, flowering induction, controlled-environment agriculture, seed yield, mini-steckling root architecture, Osmocote, multivariate analysis, photoperiod, vernalization response

## Abstract

Optimizing speed breeding protocols for biennial crops requires matching the vernalization regime with the genetic background. In this study, nine sugar beet genotypes were exposed to 12, 13, 14 or 15 weeks of vernalization and subsequently grown under controlled speed breeding conditions. Survival analysis revealed a threshold-like acceleration of bolting and flowering: 12 and 13 weeks were largely equivalent, whereas 14–15 weeks sharply increased the bolting and flowering hazard rates. Genotypic variation strongly influenced reproductive success and seed yield traits; genotype MARGARITA KWS combined early flowering with the highest seed number (361 seeds per plant) and total seed weight (5.26 g), while genotype 1K073 did not flower under any vernalization duration. A separate mini-steckling root architecture experiment with 11 genotypes showed that slow-release Osmocote fertilizer significantly increased mini-steckling fresh weight, length and width, with the strongest responses in genotypes 1K073, 1K139 and SMART LIENNA KWS. The interaction between genotype and nutrition was significant for mini-steckling fresh weight and width, indicating that optimal nutrition can modulate the expression of genotypic differences. Multivariate analyses (PCA, CVA, Mahalanobis distances) confirmed that vernalization duration had a threshold-type effect and that genotype was the dominant factor for seed traits, whereas nutrition was the main driver of mini-steckling architecture. Overall, these findings suggest that tailoring vernalization duration and nutrition to the genetic background may substantially improve the efficiency of sugar beet speed breeding.

## 1. Introduction

The transition from vegetative growth to reproductive development is a critical control point in the life cycle of higher plants, directly determining their adaptivity and reproductive success [1]. In biennial species such as sugar beet (*Beta vulgaris* L. var. *altissima*), this transition integrates a mandatory vernalization period, photoperiod perception, and a network of endogenous genetic and hormonal signals that collectively regulate bolting and flowering [2,3,4,5]. This multilayered control generates substantial genotypic variability in reproductive timing [6,7]. Synchronizing flowering across genotypes remains a major challenge for speed breeding protocols [8], limiting generation turnover in biennial crops.

The challenge of vernalization-dependent bolting is shared by many temperate biennial root vegetables [9,10,11,12]. In carrot, a network involving COL, FT, and SOC1 homologues mediates the response to flowering stimuli [13,14]. In radish, genomic studies have uncovered specific loci and candidate genes associated with differential bolting sensitivity [15]. Across biennial species, genotypic variation in vernalization requirements and photoperiod sensitivity forms a quantitative gradient rather than a simple qualitative dichotomy [16,17,18].

In sugar beet, the core module of flowering consists of an antagonistic pair of FLOWERING LOCUS T (FT) homologs: the floral repressor BvFT1 and the floral activator BvFT2 [2]. Their expression is controlled by upstream loci including *BvBTC1* and the *B2*/*BvBBX19* locus, which differentiate annual and biennial life cycles [3,19]. Additional regulators, *BvCOL1* and *BvCOL2*, also contribute to flowering time control [20]. Vernalization promotes bolting by inhibiting transcriptional repressors of *BvGI* [21]. Epigenetic mechanisms, such as DNA methylation dynamics at the shoot apical meristem, further modulate the vernalization response and contribute to genotypic differences in bolting tolerance [22]. The identification of sugar beet strains (‘BLOND’) capable of flowering under 24 h photoperiods without vernalization highlights the genetic potential to manipulate reproductive behavior in this crop [23].

Speed breeding technologies accelerate plant development through extended photoperiods, optimized light spectra, and controlled temperatures, enabling up to 4–6 generations per year in several annual crops [8,24,25,26]. In biennial crops, however, the vernalization response often saturates beyond a certain cold duration, indicating a nonlinear relationship [27]. This nonlinearity may involve a trade-off between flowering earliness and reproductive fitness, as earlier flowering can reduce seed yield and plant vigor due to resource allocation costs and developmental constraints [28,29]. Furthermore, the response of mini-steckling (a small storage root used for seed production [4]) to different nutrition regimes and its interaction with genotypes have not been investigated. These gaps highlight that optimizing speed breeding for biennial crops requires matching the vernalization duration and nutrition with the genetic background.

Building upon our previous findings on the importance of phosphorus–potassium nutrition for reproductive development [30], the present study provides a comprehensive factorial evaluation of genotype × vernalization duration and genotype × nutrition interactions under controlled speed breeding conditions. We exposed nine sugar beet genotypes to 12, 13, 14, or 15 weeks of vernalization and subsequently grew them under speed breeding conditions to record phenology and seed yield components. In a separate experiment, eleven genotypes were grown under three contrasting nutrition regimes to assess mini-steckling root architecture. We hypothesize that (i) vernalization duration exerts a nonlinear effect on reproductive timing and seed productivity, (ii) the magnitude and direction of this effect are strongly genotype-dependent, and (iii) nutrition regime modulates mini-steckling root architecture in a genotype-specific manner. By clarifying how vernalization history and nutrition interact with genetic architecture, this work aims to improve the predictability and efficiency of accelerated breeding systems and support the development of genotype-tailored protocols for sugar beet breeding and seed production.

## 2. Results

### 2.1. Phenology (Vernalization)

#### 2.1.1. General Sample Characteristics

After aggregation at the plant level, 319 plants (nine genotypes, four vernalization treatments) were included in the analysis. Bolting was recorded in 153 plants (47.9%), and flowering in 114 plants (35.7%). Genotype 1K073 did not reach any reproductive stage (40 plants, 0 events), suggesting that the applied vernalization treatments were insufficient to induce flowering in this genotype. All subsequent estimates account for the different observation periods (experiment terminated on 3 December 2025). Summary statistics for all measured traits (days to bolting, budding, lateral shoot formation, flowering, plant height and number of main flowering shoots) are provided in Table S1. The number of plants, bolting and flowering events per genotype are summarized in Table S2.

#### 2.1.2. Vernalization Duration Accelerates Bolting and Flowering Only Beyond a Threshold

Prolonged vernalization accelerated bolting and flowering, but only after a clear threshold (Table 1). Plants exposed to 12 or 13 weeks of cold treatment bolted and flowered at similar rates, with no statistically significant difference between these two durations. However, extending the vernalization to 14 or 15 weeks sharply increased the pace of development: the probability of bolting approximately tripled, and the probability of flowering more than doubled compared with 12 weeks (Table 1). The transition from 13 to 14 weeks marked a significant jump in the bolting rate, supporting a threshold-like response. The hazard ratios (HRs) reported here should be interpreted as averages over the observation period because the assumption of proportional hazards was not fully met.

Restricted mean survival time (RMST) analysis reinforced these findings (Table 2). Under the 12- and 13-week treatments, fewer than half of the plants bolted by the end of the experiment, so a median time to bolting could not be calculated. In contrast, plants that received 14 or 15 weeks of vernalization reached the median bolting time at 27 and 23 days after transplanting, respectively.

**Figure 1:**
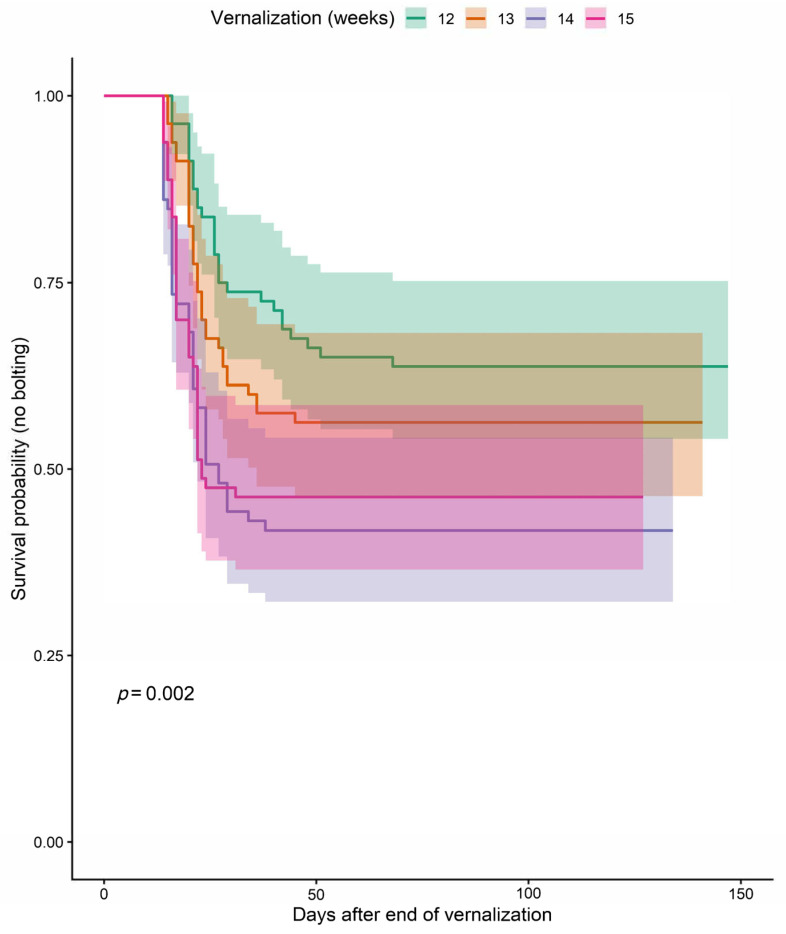
Kaplan–Meier curves for time to bolting stratified by vernalization duration (12–15 weeks). Shaded areas: 95% confidence intervals. The log-rank test *p*-value is indicated.

Kaplan–Meier curves (Figure 1) illustrate that 12 and 13 weeks of vernalization resulted in shallow and overlapping survival curves, while a clear separation emerged at 14 and 15 weeks (log-rank *p* < 0.001).

Restricted mean survival times (τ = 100 days) for time to bolting are provided in Table S3. The absolute numbers and proportions of plants reaching bolting and flowering in each vernalization duration are given in Table S4.

#### 2.1.3. Strong Genotypic Differentiation in Bolting and Flowering Propensity

Genotypes differed markedly in their bolting and flowering behavior (Table 3). Compared with the reference line 0K061, MARGARITA KWS bolted almost three times faster and flowered six times faster. In contrast, lines 1K139 and SMART LIENNA KWS bolted much more slowly, with only about one-third and one-tenth of the reference bolting rate, respectively. Genotype 1K073 never reached bolting or flowering, so its hazard ratio could not be estimated (Table S5).

**Figure 2:**
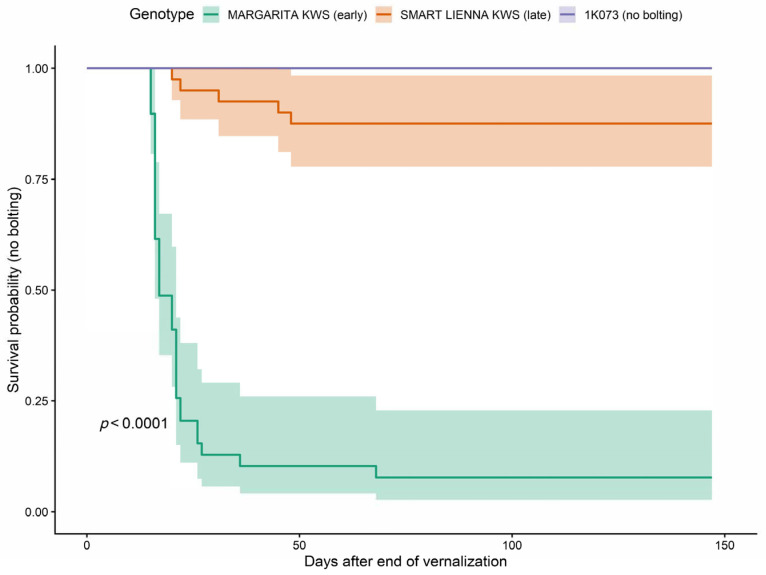
Kaplan–Meier curves for time to bolting in three representative genotypes: MARGARITA KWS (early bolting), SMART LIENNA KWS (late bolting), and 1K073 (no bolting). Data are pooled across vernalization treatments. Censoring and confidence intervals are as in Figure 1. Log-rank *p* < 0.001.

According to the estimated marginal means (EMMs) back-transformed from the log-time model, the time to bolting was 19.3 days for genotype MARGARITA KWS and 32.8 days for genotype SMART LIENNA KWS; for flowering, the corresponding EMMs were 50.2 and 71.6 days. Estimated marginal means (back-transformed from the log-time model) for days to bolting for all genotypes are given in Table S6. Estimated marginal means for days to flowering are presented in Table S7. Kaplan–Meier curves for time to flowering stratified by vernalization duration and by the three representative genotypes are shown in Figure S1. Kaplan–Meier curves for three contrasting genotypes (Figure 2) emphasize these differences.

#### 2.1.4. Agronomic Trade-Off: Total Cycle Length Increases with Longer Vernalization

**Figure 3:**
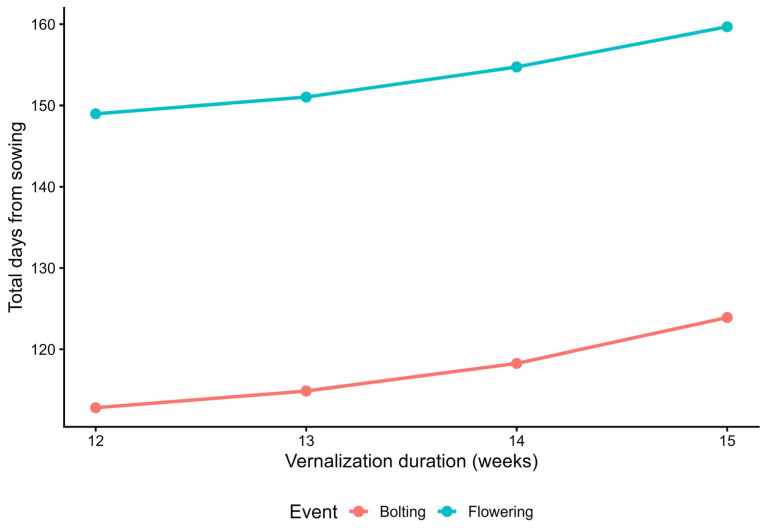
Total days from sowing to bolting (orange) and flowering (blue). Points: geometric means (±SE). The total cycle lengthens with longer vernalization despite a shorter post-vernalization phase.

When expressed as days from sowing (15 April 2025), a clear trade-off emerged (Table 4, Figure 3). The total time from sowing to bolting rose from 112.8 days (12 weeks) to 123.9 days (15 weeks); similarly, days to flowering increased from 149.0 to 159.7. Values are estimated marginal means back-transformed from the log scale and represent typical (geometric mean) times. Thus, the shortest total cycle was achieved with 12 weeks of vernalization, even though the post-vernalization phase was longer under this treatment.

#### 2.1.5. Plant Height and Number of Main Flowering Shoots

Plant height was influenced by genotype (ANOVA *p* < 0.001) but not by vernalization duration. Genotype DESIDERIA KWS reached the greatest height (90.4 cm), whereas genotype SMART LIENNA KWS was the shortest (62.0 cm). Estimated marginal means of plant height for all genotypes are presented in Table S8. The overall mean number of main flowering shoots was low (0.4 ± 0.6), reflecting the high proportion of non-bolting plants. Among the 153 plants that bolted, the average was 0.8 ± 0.58 with a median of one, demonstrating that bolted plants typically developed single main shoots.

#### 2.1.6. Sensitivity Analysis

Excluding genotype 1K073 did not alter the direction or significance of the effects (Table S5). Violations of the proportional hazards assumption were addressed by interpreting hazard ratios as time-averaged effects. A complementary time-varying coefficient Cox model (Table S9) confirmed the significant positive dependence of the bolting rate on vernalization duration (time-dependent HR = 1.12 per additional week, *p* ≈ 1 × 10^−6^), corroborating the robustness of the threshold-like pattern.

### 2.2. Seed Productivity

#### 2.2.1. Overview

Seeds were harvested from 114 plants (eight genotypes, all four vernalization treatments) that successfully flowered. Genotype 1K073 is absent, consistent with its lack of flowering. On average, a plant produced 289 ± 126 seeds (range 4–660), with a total seed weight of 4.26 ± 2.25 g (0.02–8.40 g) and a thousand-seed weight (TSW) of 14.30 ± 6.04 g (0.61–34.47 g).

#### 2.2.2. Seed Number: Genotype MARGARITA KWS Is the Most Prolific

The number of seeds per plant varied considerably among genotypes (*p* = 0.043), while the length of vernalization had no detectable influence (*p* = 0.096) (Table S10). MARGARITA KWS was the most prolific genotype, producing an average of 361 seeds per plant (range 303–430), far more than any other line. In contrast, the least productive genotype, SMART GINEVRA KWS, yielded only 134 seeds per plant (63–287) (Table 5).

#### 2.2.3. Total Seed Weight and Thousand-Seed Weight: Genotypes MARGARITA KWS and SMART DILARTA KWS Stand out

Total seed weight and TSW were strongly genotype-dependent (*p* < 0.001) but were not influenced by vernalization duration (Table 5 and Table S10). Genotype MARGARITA KWS had the highest total seed weight (5.26 g, CI 4.40–6.30), whereas genotypes SMART GINEVRA KWS and 1K139 gave the lowest yields (1.69 g and 1.76 g). Genotype SMART DILARTA KWS produced the largest seeds (TSW = 16.46 g, CI 14.01–19.33).

**Figure 4:**
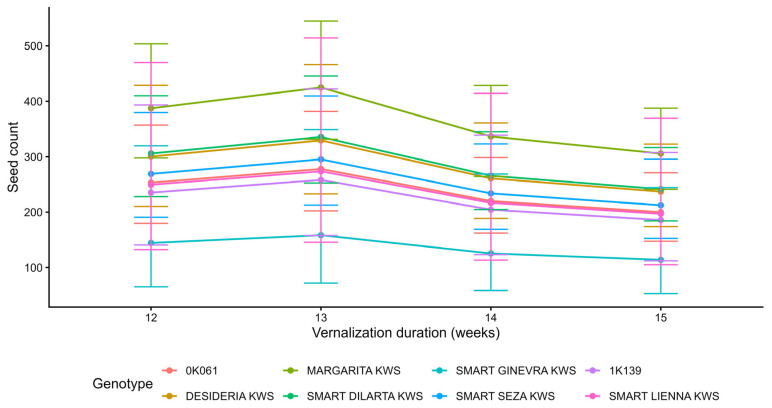
Estimated marginal means for seed number per plant across vernalization durations (negative binomial GLM). Error bars: 95% confidence intervals. MARGARITA KWS produces the highest seed number.

**Figure 5:**
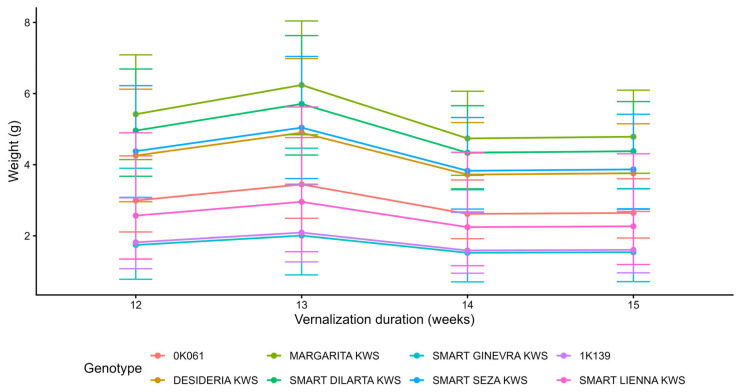
Estimated marginal means for total seed weight per plant (Gamma GLM with log-link). Error bars: 95% confidence intervals. MARGARITA KWS maintains high seed weight across treatments.

A weak but significant genotype × vernalization duration interaction was detected for total seed weight (*p* = 0.017) (Table S11). Interaction plots (Figure 4 and Figure 5) confirm that genotype MARGARITA KWS maintained high productivity across all vernalization treatments. Estimated marginal means for each vernalization duration, confirming the absence of a main effect of this factor, are reported in Tables S12 (seed number), S13 (total seed weight) and S14 (thousand-seed weight).

#### 2.2.4. Non-Parametric Validation and Model Diagnostics

**Figure 6:**
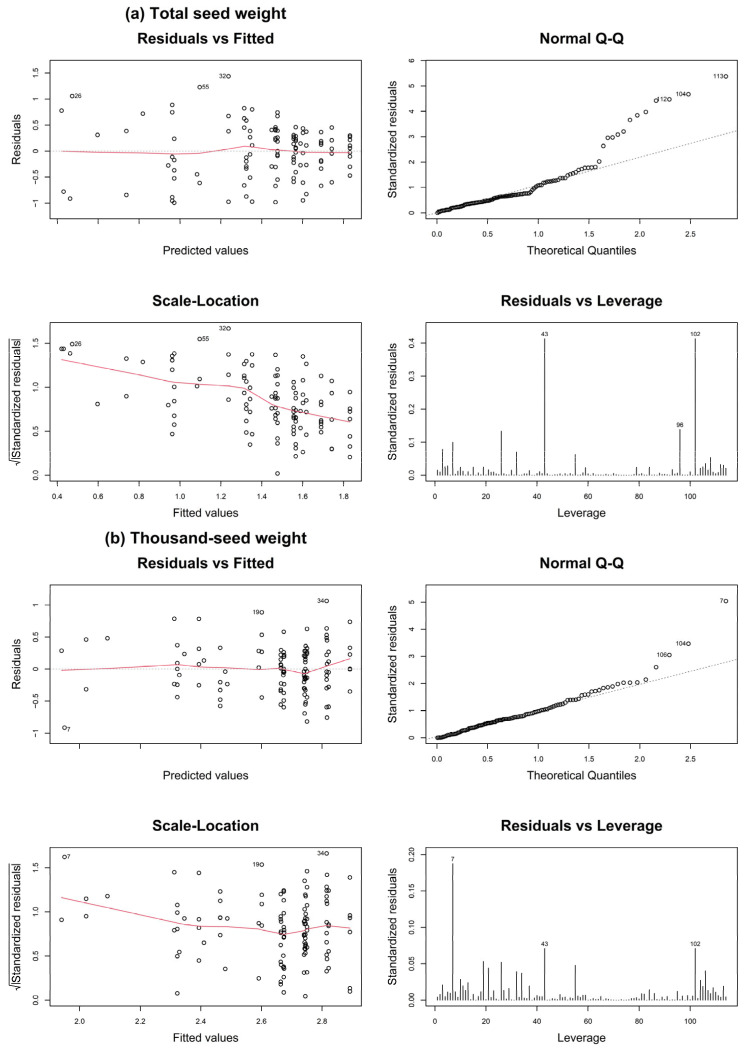
Diagnostic plots for the Gamma GLMs of (**a**) total seed weight and (**b**) thousand-seed weight. Standard four-plot suite: residuals vs. fitted, Q-Q, scale-location, residuals vs. leverage. Circles represent individual observations; solid red lines are LOESS (locally estimated scatterplot smoothing) curves that show the trend of the residuals; dashed lines mark zero-residual, theoretical normal, or equal Cook’s distance contours as appropriate for each panel.

Kruskal–Wallis tests confirmed the significant effect of genotype on all three traits (*p* < 0.01). Vernalization duration was not significant (*p* > 0.1). Diagnostic plots for the gamma models (Figure 6) showed no systematic problems.

### 2.3. Mini-Steckling Root Architecture

#### 2.3.1. Overview

A total of 247 mini-stecklings (11 genotypes, three nutrition regimes) were analyzed. The measured traits were mini-steckling fresh weight (g), length (cm) and width (cm).

#### 2.3.2. Mini-Steckling Fresh Weight: Osmocote Is the Best Promoter, with Genotype 1K073 Among the Top Performers

Nutrition and genotype both affected mini-steckling fresh weight, and their interaction was also significant (*p* < 0.001 for all effects) (Table 6). Mini-stecklings supplied with Osmocote were, on average, 1.6 times heavier than those receiving only liquid fertilizer, with mean fresh weights of 34.7 g, 21.8 g, and 20.1 g for Osmocote, control, and liquid supplement, respectively (Table 7).

Averaged across nutrition regimes, the highest mini-steckling fresh weight was produced by genotypes 1K073, 1K139 and SMART LIENNA KWS (28.6, 28.3 and 27.6 g, respectively). However, genotypic differences were not detectable without accounting for nutrition and its interaction (marginal Kruskal–Wallis test: *p* = 0.163).

**Figure 7:**
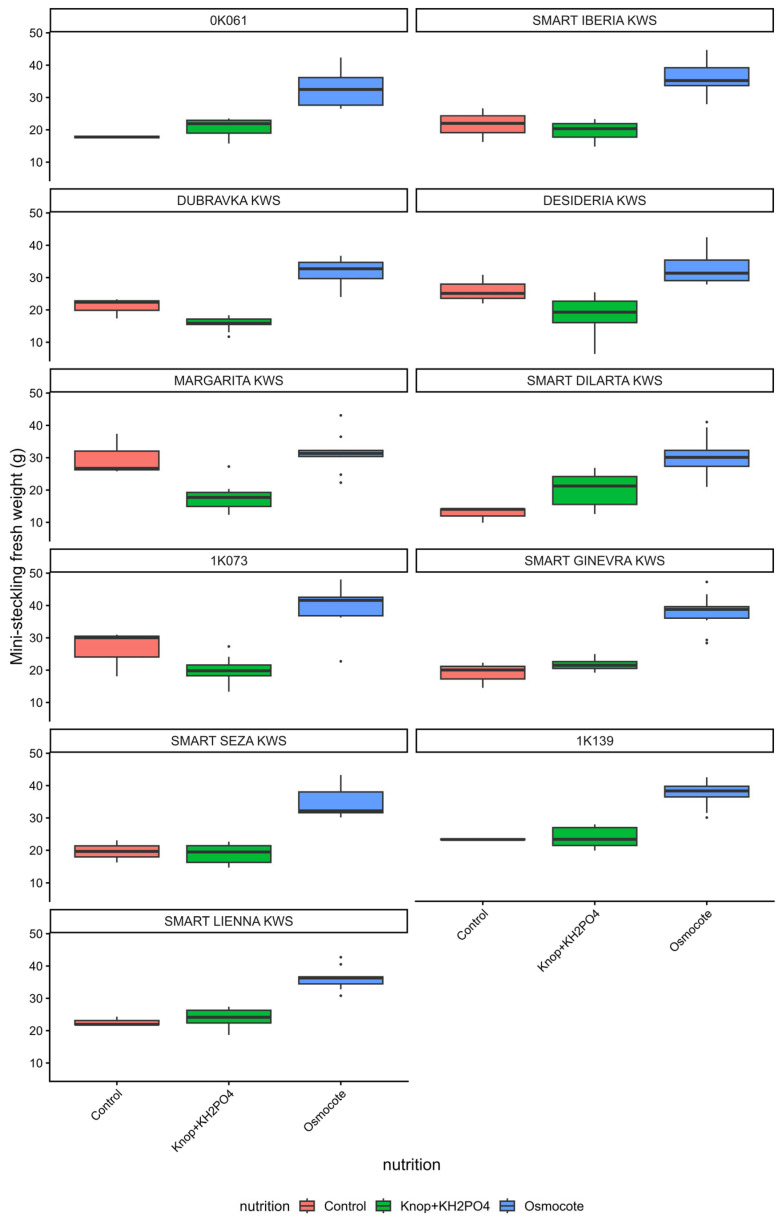
Boxplots of mini-steckling fresh weight (g) by nutrition regime and genotype. Boxes: IQR; whiskers: 1.5 × IQR; horizontal line: median. Osmocote strongly promotes mini-steckling fresh weight.

In most lines, Osmocote significantly increased mini-steckling fresh weight relative to both the control and the liquid supplement (*p* < 0.001) (Table S15). The exceptions were genotypes DESIDERIA KWS and MARGARITA KWS, where the Osmocote effect was less pronounced. Boxplots of mini-steckling fresh weight for each genotype × nutrition combination are shown in Figure 7.

**Figure 8:**
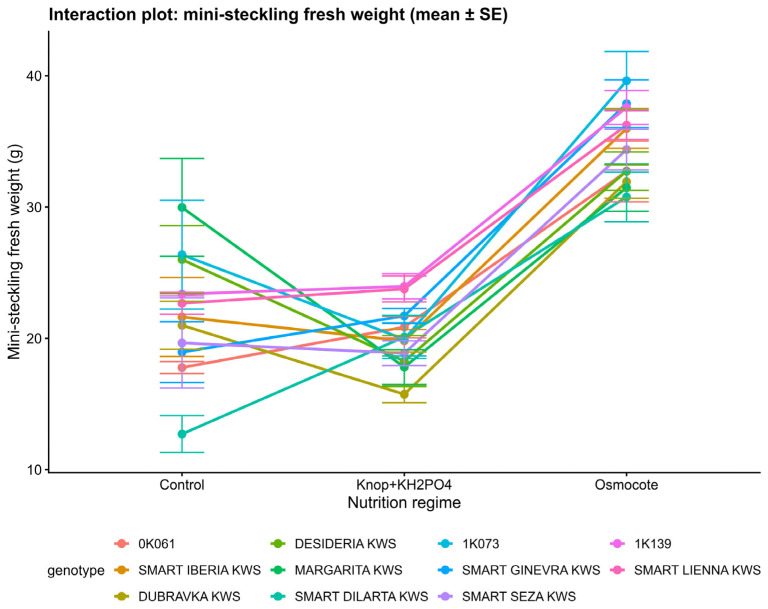
Interaction plot of mini-steckling fresh weight (mean ± SE) for genotype–nutrition combinations. Error bars: ±1 standard error. Genotypes differ markedly in their response to Osmocote.

When grown with Osmocote, genotype 1K073 (39.6 g) significantly outperformed many other lines, whereas genotype SMART DILARTA KWS (30.8 g) was among the lowest (Table S16). An interaction plot of raw means for mini-steckling fresh weight is given in Figure 8.

#### 2.3.3. Mini-Steckling Length: Only Nutrition Matters

**Figure 9:**
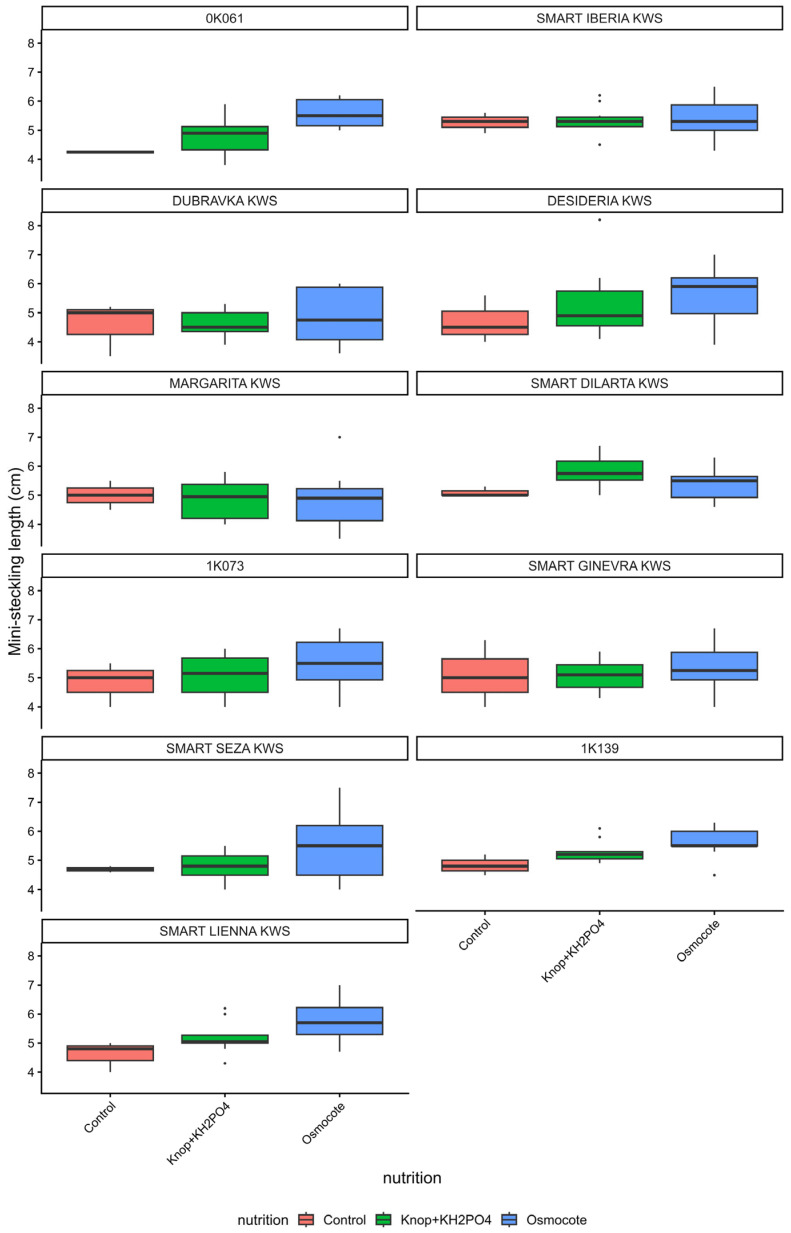
Boxplots of mini-steckling length (cm) by nutrition regime and genotype. Construction as in Figure 7. Nutrition effect is small but statistically significant. Black dots beyond the whiskers are individual data points lying outside 1.5 × IQR (outliers).

Mini-steckling length was affected solely by nutrition (*p* = 0.026) (Table 6). Osmocote produced the greatest mean length (5.39 cm) (Table 7). A non-parametric Kruskal–Wallis test detected significant differences among genotypes (*p* = 0.0055) (Table S17), indicating that marginal genotypic variation exists but is largely overridden when nutrition is included in the two-way model. Boxplots of mini-steckling length are presented in Figure 9.

#### 2.3.4. Mini-Steckling Width: Osmocote Expands the System, Genotype 1K073 Leads

Mini-steckling width was influenced by nutrition, genotype and their interaction (*p* = 0.022, *p* = 0.00031 and *p* = 0.011) (Table 6). Osmocote increased the average width to 36.3 cm (Table 7). The widest mini-stecklings were developed by genotypes 1K073 (33.5 cm), SMART LIENNA KWS (33.6 cm) and DESIDERIA KWS (32.3 cm).

**Figure 10:**
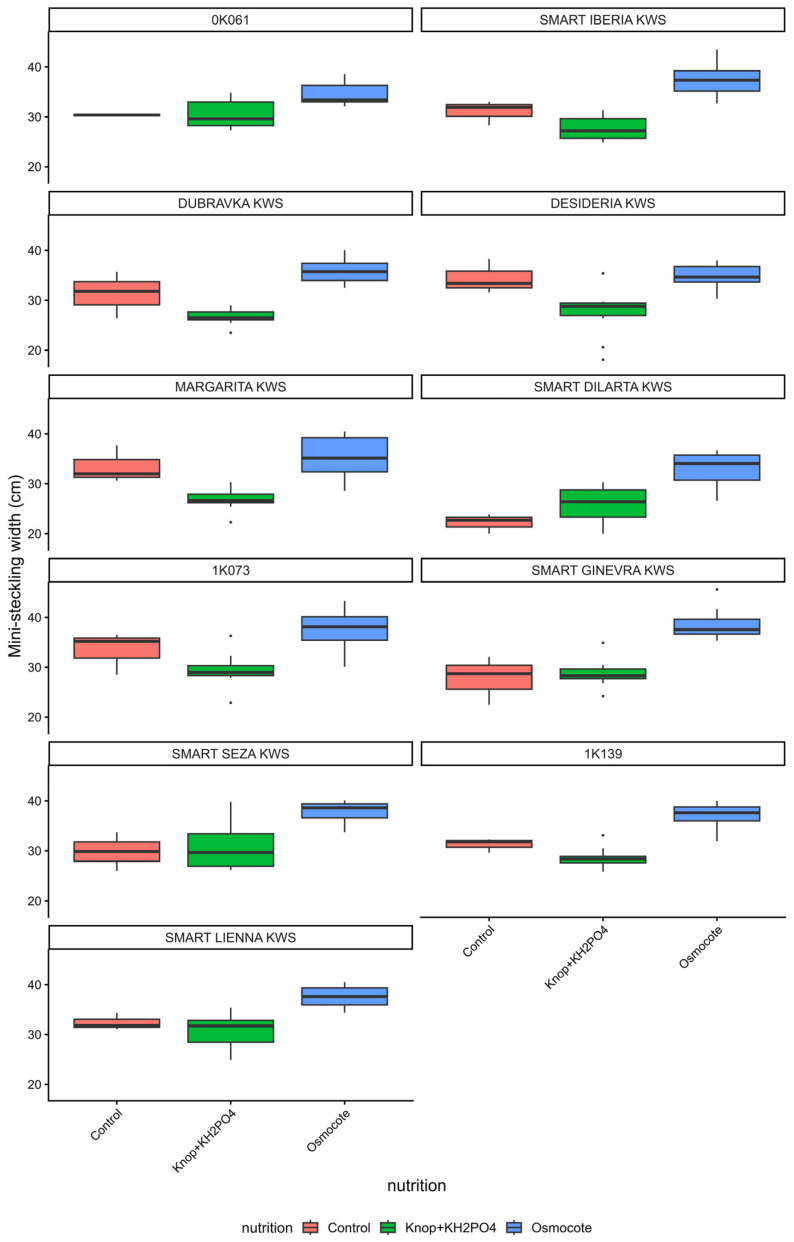
Boxplots of mini-steckling width (cm) by nutrition regime and genotype. Construction as in Figure 7. Osmocote sharply increases mini-steckling width. Black dots beyond the whiskers are individual data points lying outside 1.5 × IQR (outliers).

Simple-effect analyses (Tables S18 and S19) confirmed that Osmocote significantly increased mini-steckling width in most genotypes, with the exception of DESIDERIA KWS and MARGARITA KWS where the effect was attenuated. Boxplots of mini-steckling width are shown in Figure 10.

#### 2.3.5. Robustness of the Findings

Non-parametric Kruskal–Wallis tests (Table S17) confirmed the highly significant effect of nutrition (*p* < 0.001). The marginal genotype effect was not significant (*p* = 0.163), reinforcing the conclusion that genotypic effects on mini-steckling fresh weight are expressed primarily through their interaction with nutrition. Diagnostic residual plots for the mini-steckling models are provided in Figures S2–S4.

### 2.4. Multivariate Analysis

To complement the univariate analyses, we performed principal component analysis (PCA) (Tables S20–S22; HTML Figures S5–S13), canonical variate analysis (CVA) (Tables S23 and S24; HTML Figures S14–S19), calculated Mahalanobis distances (Table S25) and MANOVA results (Pillai’s trace) (Table S26) for each of the three data blocks (phenology, seed traits, mini-steckling root architecture). As the three experiments involved different sets of plants, different trait batteries, and distinct treatment designs, Mahalanobis distances were calculated within each multivariate space separately to maintain biological and statistical coherence.

Both Spearman rank (Table S27) and Pearson correlations (Table S28) were computed; results were qualitatively similar, and Spearman coefficients are reported throughout because of their robustness to outliers and non-normality. PCA was used to explore the overall correlational structure without imposing group labels, whereas CVA maximized the separation among predefined groups (genotype, vernalization duration, or nutrition), thereby accounting for within-group variability and providing a direct test of group differences.

#### 2.4.1. Phenology

PCA confirmed that bolting and flowering times were closely linked (Spearman ρ = 0.71). The first two components captured 68% of the variance: the first (42% of variation) separated plants primarily by how quickly they bolted and flowered, while the second (26%) was associated with plant height and shoot number. A full-factorial MANOVA (genotype × vernalization) confirmed significant effects of genotype (Pillai’s trace = 1.21, *p* < 0.001), vernalization duration (Pillai’s trace = 0.46, *p* < 0.001), and their interaction (Pillai’s trace = 0.93, *p* = 0.018) (Table S26). The Mahalanobis distance between the centroids of the 14- and 15-week groups was negligible (0.12), whereas both were clearly separated from the 12- and 13-week groups (distances 1.23–1.26), quantitatively supporting the threshold-like vernalization response. Pairwise MANOVAs confirmed that the 12- and 13-week groups did not differ significantly (*p* = 0.19), nor did the 14- and 15-week groups (*p* = 0.99), whereas all comparisons between the 12–13-week cluster and the 14–15-week cluster were significant or approached significance (*p* ≤ 0.087; full results in Table S29). Linear discriminant analysis (LDA) correctly classified 33.3% of the plants by genotype and 47.4% by vernalization duration (Table S30).

#### 2.4.2. Seed Traits

For seed traits, the first two principal components captured almost all the variability (98.9% of the total variance). The first component (71.6%) could be interpreted as overall seed productivity, with high values for plants producing many and heavy seeds. The second component (27.3%) revealed a trade-off between seed number and seed size. Genotypes MARGARITA KWS and SMART DILARTA KWS were both highly productive, but SMART DILARTA KWS tended to produce fewer, larger seeds, consistent with its high thousand-seed weight. Importantly, vernalization duration did not separate the genotypes in the multivariate space, and its correlations with seed traits were weak (all |ρ| < 0.30). A full-factorial MANOVA (genotype × vernalization) confirmed a significant genotype effect (Pillai’s trace = 0.43, *p* = 0.006) and a weaker but significant vernalization effect (Pillai’s trace = 0.25, *p* = 0.009), with no interaction (*p* = 0.96) (Table S26). CVA loadings reinforced this picture: the first canonical variate was heavily loaded on total seed weight and seed number; the second reflected the size-versus-number trade-off. LDA accuracy was 29.8% for genotype and 35.1% for vernalization duration (Table S30).

#### 2.4.3. Mini-Steckling Root Architecture

The first two PCA dimensions of root traits explained 97.4% of the total variance. Fresh weight and width were strongly associated (angle between vectors = 28°), whereas root length varied almost independently of them (angles > 75°). A full-factorial MANOVA (nutrition × genotype) confirmed significant effects of nutrition (Pillai’s trace = 0.78, *p* < 0.001), genotype (Pillai’s trace = 0.49, *p* < 0.001), and their interaction (Pillai’s trace = 0.36, *p* = 0.016) (Table S26). When nutrition was used as the grouping factor, Osmocote-treated plants formed a distinct cluster well separated from the other two regimes; the multivariate distances between Osmocote and the control or liquid supplement were large (1.51 and 1.68), whereas the control and liquid supplement overlapped substantially (distance 0.57). Pairwise MANOVAs confirmed that Osmocote differed strongly from both the control (*p* < 2 × 10^−16^) and the Knop + KH_2_PO_4_ regime (*p* < 2 × 10^−53^), whereas control and Knop + KH_2_PO_4_ overlapped considerably (*p* = 0.016; Table S29). Linear discriminant analysis classified 75.7% of the plants correctly by nutrition, but only 20.2% by genotype, confirming that nutrition is the primary driver of root architecture while genotypic differences are detectable but secondary (Table S30). Among genotypes, the separation was moderate, with the largest differences involving SMART SEZA KWS and SMART DILARTA KWS.

## 3. Discussion

The present study provides a comprehensive evaluation of genotype × vernalization duration interactions for reproductive timing, seed productivity and mini-steckling root architecture in sugar beet under speed breeding conditions. Three principal findings emerge: (i) vernalization duration accelerates bolting and flowering in a distinctly threshold-like manner, with 14 and 15 weeks being largely equivalent and clearly separated from 12 and 13 weeks; (ii) genotypic variation dominates seed yield components, with MARGARITA KWS and SMART DILARTA KWS forming a high-productivity cluster while SMART GINEVRA KWS and 1K139 remain low-yielding, and vernalization duration has no appreciable multivariate effect on seed traits; and (iii) mini-steckling root architecture responds strongly to slow-release Osmocote fertilization, and genotype × nutrition interactions are significant for mini-steckling fresh weight and width, whereas mini-steckling length varies largely independently of the other two dimensions.

### 3.1. Vernalization Exerts an Apparent Threshold-like Effect on Bolting and Flowering

Cox regression and RMST analyses concordantly showed that 12 and 13 weeks of vernalization were largely equivalent. A marked acceleration in bolting and flowering occurred only at 14 and 15 weeks. This pattern fits the vernalization-intensity model developed for sugar beet by Milford et al. [27], which describes the proportion of bolted plants as a function of accumulated vernalizing hours above a genotype-specific threshold (VR). In our experiment, the constant temperature of +4 °C falls close to the optimum of the vernalization weighting curve [27]; therefore, the duration of cold exposure can be regarded as a proxy for the intensity of vernalization. The median bolting time was reached only after 14 and 15 weeks. Moreover, the Mahalanobis distance between the 14- and 15-week centroids was negligible (0.12), while both groups were clearly separated from the 12- and 13-week groups (distances of 1.23–1.26). Together, these results provide quantitative, multivariate support for threshold-like behavior. MANOVA corroborated this pattern: the centroids of the 14-week and 15-week groups did not differ significantly, whereas both differed from the 12-week and 13-week groups.

Rather than a strict binary switch, this pattern reflects an “apparent threshold” that arises from the requirement to accumulate a sufficient duration of low-temperature exposure to stably repress floral inhibitors. In sugar beet, the core regulatory module is centered on the antagonistic pair BvFT1 (repressor) and BvFT2 (activator), which are controlled by upstream components such as *BvBTC1* and the *B2/BvBBX19* locus [2,3]. Cold exposure progressively down-regulates *BvFT1* and up-regulates *BvFT2*, ultimately tipping the balance toward flowering [2]. The steep increase in flowering rate between 13 and 14 weeks is consistent with vernalization-intensity models developed for sugar beet, which predict that a minimum number of vernalizing hours must be exceeded before bolting is initiated [27]. Epigenetic modifications at the shoot apical meristem, such as DNA methylation changes documented during vernalization in beet [22], may further reinforce this vernalized state.

A molecular framework for this threshold is offered by the recent work of Zhang et al. [21], who demonstrated that vernalization progressively represses the transcriptional inhibitors of *BvGI* (*BvLHY*, *BvTCP4* and *BvCRF4*), leading to a continuous up-regulation of *BvGI* from 8 to 16 weeks of cold treatment. Our 14- and 15-week treatments likely exceed the point at which the repressors are sufficiently silenced and BvGI (and possibly downstream activators such as BvFT2) reaches the level required to commit the meristem to flowering. The similarity of the 14- and 15-week responses suggests that once this molecular switch is thrown, additional cold exposure provides little further acceleration of the bolting rate. This is fully consistent with the vernalization-intensity concept of a critical threshold, beyond which the system saturates [21,27].

### 3.2. Genotypic Variation Dominates Reproductive Success and Seed Output

Among the eight genotypes that flowered, seed yield traits were overwhelmingly determined by genotype, as shown by both univariate models and the CVA/Mahalanobis analysis. The first two principal components of the seed-trait PCA captured 98.9% of the total variance: PC1 was an overall productivity axis, while PC2 revealed a trade-off between seed number and thousand-seed weight. MARGARITA KWS and SMART DILARTA KWS occupied the high-productivity end of PC1, yet differed in PC2: SMART DILARTA KWS showed a clear shift toward larger seeds, consistent with its highest thousand-seed weight. This genetic variation in seed size is consistent with the study of Yousefabadi and Rajabi [31], where significant additive variance and high narrow-sense heritability (0.84) for 1000-germ weight in sugar beet are reported, indicating that seed size can be effectively improved by selection. CVA loadings reinforced this interpretation (Section 2.4).

Importantly, neither PCA nor CVA revealed any appreciable effect of vernalization duration on the multivariate seed phenotype, and Spearman correlations between vernalization duration and the three seed traits were weak (all |ρ| < 0.30). A weak but significant interaction between genotype and vernalization duration was detected for total seed weight (*p* = 0.017). This indicates that some genotypes may adjust how resources are allocated to seed filling depending on the length of cold treatment, although the effect is small compared with overall genotypic differences. Because only plants that flowered contributed to the seed dataset, some of the vernalization effect may have been filtered out at the bolting and flowering stages.

### 3.3. Trade-Off Between Cycle Length and Flowering Synchrony

Extending vernalization from 12 to 15 weeks increased the total time from sowing to flowering, because the extra cold period outweighed the shorter post-vernalization phase. Thus, for maximizing the number of generations per year, 12 weeks of vernalization is optimal. For maximizing the synchrony of flowering for controlled crosses, 14–15 weeks may be preferable, as they substantially increase the proportion of bolted plants and reduce the variability in bolting time. This trade-off is similar to that faced in autumn-sown sugar beet production, where early sowing increases yield potential but also bolting risk [27,32]. The vernalization-intensity model, parameterized with variety-specific VR and bolting sensitivity, can help breeders to choose the optimal vernalization duration for a given set of genotypes, especially when the aim is to balance generation turnover and synchrony in speed breeding pipelines.

### 3.4. Mini-Steckling Root Architecture Is Shaped by Nutrition and Genotype–Nutrition Interplay

Osmocote consistently promoted mini-steckling fresh weight, length and width, with the strongest responses in genotypes 1K073, 1K139 and SMART LIENNA KWS. The PCA of mini-steckling traits showed that mini-steckling fresh weight and width were tightly associated (angle between vectors = 28°), whereas mini-steckling length was nearly orthogonal to both (angles ≈ 75° and 90°). This indicates that, under the applied nutritional regimes, elongation responded to treatments largely independently of radial expansion and biomass accumulation. Stevanato et al. [33] also identified root elongation rate as a strongly genotype-dependent trait correlated with productivity. Our data extend these findings by showing that genotypic differences in mini-steckling fresh weight and width become apparent only when the nutritional background is taken into account.

The significant genotype × nutrition interaction for mini-steckling fresh weight and width was mainly driven by the fact that genotypes DESIDERIA KWS and MARGARITA KWS showed a weaker response to Osmocote than the other lines. The molecular or physiological basis of this differential response remains to be elucidated, but it may be related to genotypic differences in nutrient uptake efficiency, hormone signaling or root architecture plasticity [33]. A simple Kruskal–Wallis test ignoring nutrition failed to detect genotypic differences in mini-steckling fresh weight (*p* = 0.163). However, when nutrition and its interaction with genotype were included in the two-way ANOVA, the genotype effect became highly significant. The dominance of nutrition over genotype was underlined by linear discriminant analysis: 75.7% of plants were correctly assigned to their nutritional regime, compared with only 20.2% for genotype. This contrast illustrates that genotypic variation in mini-steckling fresh weight is masked unless the nutritional environment is explicitly taken into account. This has practical implications for speed breeding: optimizing nutrition can partially offset genotype-specific limitations and lead to a more uniform and vigorous root system, which may in turn support better shoot growth and seed production.

### 3.5. Toward an Integrated Physiological Perspective

Although the three experimental blocks were largely analyzed separately, they are physiologically linked. Our previous work on phosphorus–potassium nutrition [30] demonstrated that balanced P and K supply is critical for sugar beet reproductive development. The slow-release formulation of Osmocote PRO (18-9-10 + 2MgO) supplies P and K steadily across growth stages. This favors the formation of larger mini-stecklings, which—after cold storage and transplantation—can support more robust seed production. Independent observations in steckling-based systems are consistent with this pattern [34]. Improved mini-steckling root biomass under Osmocote may enhance nutrient and water uptake, supporting greater plant vigor and potentially more robust reproductive development. In the present dataset, however, mini-steckling traits averaged per genotype under Osmocote were negatively correlated with seed yield components across the eight genotypes common to both experiments. For example, mini-steckling fresh weight and width showed a strong inverse relationship with seed number (Spearman ρ = −0.74 and −0.71, respectively) (Table S31); Pearson correlations gave consistent results (Table S32). This points to a resource–allocation trade-off between storage root biomass and reproductive output, in line with the hormonal modularity framework [35]. This inverse association likely reflects genotypic differences in resource allocation between vegetative (mini-steckling) and reproductive organs, rather than a direct physiological link within the same plant, since mini-steckling and seed data were obtained from separate experiments. This pattern is consistent with the plant hormone modularity framework [5,35], where a trade-off exists between reproduction maximization (driven by cytokinins and gibberellins) and reproduction assurance (driven by auxins, ABA and stress hormones). Genotypes that develop a larger mini-steckling may be skewed toward the survival-assurance module, which curtails reproductive output, whereas high-seed-yielding genotypes like MARGARITA KWS appear to prioritize the cytokinin-mediated reproduction maximization program. Dedicated experiments in which mini-steckling architecture and seed production are recorded on the same individuals would be necessary to rigorously test the hypothesized positive feedback between a vigorous root system and reproductive output under speed breeding conditions. Genotypes that combine a strong mini-steckling response to nutrition with a moderate vernalization requirement (e.g., genotype MARGARITA KWS under optimal nutrition) may represent ideal candidates for accelerated breeding pipelines.

### 3.6. Limitations and Outlook

In addition to the limitations already discussed (absence of a non-vernalized control, uncharacterized molecular background of genotype 1K073, controlled-environment conditions, and potential inflation of Type I error due to multiple testing), it should be noted that we expressed vernalization duration in calendar weeks for practical clarity. Translating these durations into thermal-time vernalization doses, as proposed by Milford et al. [27] and used by Sadeghzadeh Hemayati et al. [32] and in our previous work [30], would facilitate comparisons across studies and help refine genotype-specific cold requirements. Future work should also test whether recently identified molecular markers (e.g., *BvLHY*, *BvTCP4*, *BvCRF4*; [21]) can predict vernalization sensitivity in breeding material. Similarly, AFLP markers associated with root elongation rate [33] could be deployed to select for improved mini-steckling root systems under controlled nutrition. Despite these caveats, this study demonstrates that integrating genotype, vernalization duration and nutrition is essential for efficient speed breeding in sugar beet. We cannot exclude that genotypic differences in photoperiod sensitivity, in addition to the vernalization requirement, contributed to the observed variation in flowering, especially for line 1K073, which remained vegetative under all treatments [20,23].

## 4. Materials and Methods

### 4.1. Plant Material

Certified seeds of eleven sugar beet (*Beta vulgaris* L. var. *altissima*) hybrids developed by KWS (Einbeck, Germany) were used. The eight named varieties (DESIDERIA KWS, MARGARITA KWS, SMART LIENNA KWS, SMART DILARTA KWS, SMART GINEVRA KWS, SMART SEZA KWS, SMART IBERIA KWS, and DUBRAVKA KWS) are registered commercial F_1_ hybrids. The designations 0K061, 1K073, and 1K139 refer to experimental breeding lines not commercially released. For brevity, all entries are collectively referred to as “genotypes” throughout the text. The nine genotypes employed for phenological observations and seed productivity assessment were: 0K061, DESIDERIA KWS, MARGARITA KWS, SMART DILARTA KWS, 1K073, SMART GINEVRA KWS, SMART SEZA KWS, 1K139, and SMART LIENNA KWS. The mini-steckling root architecture experiment additionally included two further commercial hybrids: SMART IBERIA KWS and DUBRAVKA KWS.

### 4.2. Seed Pretreatment

Seeds were rinsed under running tap water at ambient temperature for 30 min, subsequently treated for 30 min with the fungicide “Maxim Dachnik” (Syngenta Crop Protection SA, Saint-Sauveur, France) at a concentration of 2 mL formulation per liter of water, and then air-dried on sterile filter paper [30].

### 4.3. Experiment 1. Effect of Vernalization Duration on Phenology and Seed Productivity

#### 4.3.1. Experimental Design and Growth Conditions

Sowing took place on 15 April 2025. Seeds were sown individually at a depth of 0.5 cm in 264-cell plug trays (cell volume 10 mL) filled with neutralized raised-bog peat “Agrobalt-S” (Pindstrup LLC, Mytishchi, Russia). Immediately after sowing, the trays were placed in a controlled-environment chamber (phytotron) where germination and vernalization occurred simultaneously. Vernalization conditions were as follows: photoperiod 22 h light/2 h dark, mean temperature +4 °C, relative air humidity 70%, and white-LED illumination providing an average PPFD of 60 µmol·m^−2^·s^−1^. The temperature of +4 °C was chosen because it is close to the optimum of the vernalization weighting curve [27], and the range of 12–15 weeks follows standard protocols for sugar beet speed breeding [4].

The cold period lasted 12, 13, 14 or 15 weeks from the sowing date. Immediately after the completion of vernalization (on 9 July, 15 July, 22 July and 29 July 2025, respectively), seedlings were transplanted with the root ball into 800 mL pots filled with a mixture of peat, sand, vermiculite, agroperlite and diatomaceous earth. Controlled-release fertilizer Osmocote Pro 8–9M (ICL Group, Amsterdam, The Netherlands) granules were incorporated into the substrate at transplanting at a rate of 1.6 g per pot.

Transplanted plants were transferred to a growth room providing speed breeding conditions: photoperiod 22/2 h, day/night temperatures +22 °C/+20 °C, and humidity 50%. Illumination was supplied by white-LED lamps supplemented with far-red light; the average photosynthetically active radiation PPFD = 195 µmol·m^−2^·s^−1^ and total photon flux density PFD = 226 µmol·m^−2^·s^−1^. These conditions follow the general speed breeding protocol [26] and were adapted for sugar beet, as described in [4]. Plants were irrigated with settled tap water applied directly to the root zone as needed. From the moment bolting was recorded, watering was supplemented with K_2_HPO_4_ solution at the concentration used in Knop’s solution (0.25 g·L^−1^) [30].

Initially, ten plants were assigned to each genotype × vernalization duration combination (the total number of plants included in the phenology analysis was 319). The experimental design was fully factorial. No plants were excluded from the analysis due to mortality or abnormal growth.

#### 4.3.2. Phenological Observations

Plants were inspected daily to record the dates of the following developmental stages using a standardized developmental scale: bolting—elongation of a flowering main stem; budding—appearance of the first visible flower buds; emergence of lateral flowering shoots; flowering—opening of the first flower; and drying—cessation of watering when seed stalks turned brown and started to desiccate, with fruit set visible [36]. Time to each stage was calculated from the end of vernalization (the day of transplanting). Plants that had not attained a given stage by the censoring date (3 December 2025) were treated as right-censored.

After the completion of flowering, plant height (from the substrate surface to the apex of the tallest generative shoot) was measured and the number of main flowering shoots was counted.

#### 4.3.3. Seed Productivity Assessment

Seeds were harvested individually from every plant that reached full maturity. Seed capsules were collected manually from each shoot, sieved through laboratory sieves with mesh sizes of 4.5 and 3 mm to remove debris and floral remains, weighed and counted. Total seed number, total seed weight and thousand-seed weight were determined for each plant [31]. Thousand-seed weight was calculated as (total seed weight/seed number) × 1000. The final analysis of seed traits included 114 plants (eight genotypes; hybrid 1K073 did not produce flowering plants under any vernalization duration).

### 4.4. Experiment 2. Mini-Steckling Root Architecture Under Different Nutritional Regimes

#### 4.4.1. Experimental Design

The experiment was conducted on mini-stecklings of the 11 genotypes listed in Section 4.1. Sowing was performed on 16 April 2025. Plants were grown in 800 mL pots containing the same substrate mixture as in Experiment 1, but without addition of Osmocote at the outset. Until day 38 after sowing, all plants received identical care. Plants were cultivated in a growth room under speed breeding conditions: photoperiod 22/2 h, day/night temperatures +22 °C/+20 °C, and humidity 50%. Illumination was provided by white-LED lamps with an average PPFD of 220 µmol·m^−2^·s^−1^. Irrigation was performed with settled tap water applied directly to the root zone as required [30].

On day 38, three contrasting nutritional regimes were established:

- Osmocote (slow-release)—Osmocote Pro 8–9M granules were incorporated once into the substrate at 1.6 g per pot (106 plants) [37].
- Control (Knop’s solution)—Weekly watering with 50 mL of Knop’s solution per pot (31 plants). Composition of Knop’s solution (per liter): Ca(NO_3_)_2_ 1 g, MgSO_4_ 0.25 g, K_2_HPO_4_ 0.25 g, KCl 0.125 g, and FeSO_4_ 0.125 g [38].
- Enhanced phosphorus–potassium nutrition (Knop + KH_2_PO_4_)—On day 38, watering with 50 mL of Knop’s solution was immediately followed by the application of 30 mL of KH_2_PO_4_ solution (40 g·L^−1^ tap water); this procedure was repeated weekly (110 plants) [30].

The actual number of replicates per genotype is given in Table 8.

#### 4.4.2. Mini-Steckling Trait Measurement

Eighty-five days after sowing, plants were carefully removed from the pots and the mini-stecklings were thoroughly washed free of substrate. After removing excess moisture with filter paper, the following parameters were recorded, mini-steckling fresh weight (g), mini-steckling length (cm), and maximum projected width of the mini-steckling (cm), measured with an electronic caliper [39].

### 4.5. Characteristics of Osmocote Pro 8–9M Fertilizer

The fertilizer (ICL Group, Amsterdam, The Netherlands) contains (%): total N 18 (including nitrate N 6.6, ammonium N 9, urea 1.4); P_2_O_5_ 9; water-soluble K_2_O 10; MgO_2_; and micronutrients—B 0.02, Cu 0.037, Fe 0.33, Mn 0.04, Mo 0.015, and Zn 0.011.

### 4.6. Statistical Analysis

All analyses were performed in R (version 4.5.3) [40]. Survival analysis was conducted using Cox proportional hazards models (standard and stratified by genotype) to estimate hazard ratios for bolting and flowering [41]; the proportional hazards assumption was checked with Schoenfeld residuals. Restricted mean survival time (RMST) was calculated with τ = 100 days [42]. Plant height was analyzed by one-way ANOVA; the number of main flowering shoots was analyzed by the Kruskal–Wallis test [43]. Seed number was modeled with a negative binomial generalized linear model (GLM) [44], while total seed weight and thousand-seed weight were modeled with Gamma GLMs (log-link) [44]; Type III ANOVA was obtained using the car package [45]. Mini-steckling traits were analyzed by two-way ANOVA (Type III SS) with simple effects examined where the nutrition × genotype interaction was significant. Non-parametric Kruskal–Wallis tests complemented the parametric analyses [43].

Multivariate analyses included principal component analysis (PCA) [46], canonical variate analysis (CVA) based on linear discriminant analysis [47] as implemented in the MASS package [44], and pairwise Mahalanobis distances [48] calculated from the within-group covariance matrix. Interactive 3D and 2D plots were generated with the plotly package [49].

The analyses relied on the R packages tidyverse, survival [41], survminer, emmeans [50], car [45], MASS [44], broom and plotly [49].

All tests were two-sided; *p* < 0.05 was considered significant. The full analysis code and output files are available as online Supplementary Materials. In all regression models, genotype 0K061 and vernalization duration of 12 weeks were used as reference levels for the respective factors.

For ANOVA models, the normality of residuals was checked with the Shapiro–Wilk test and homogeneity of variances with Levene’s test. When minor deviations occurred, non-parametric Kruskal–Wallis tests were used to confirm the results. For MANOVA, Pillai’s trace was chosen because it is robust to violations of multivariate normality and homogeneity of covariance matrices.

Correlation analyses were performed using both Spearman’s rank correlation and Pearson’s product-moment correlation. Spearman coefficients are reported in the main text because they do not require normally distributed data and are less sensitive to outliers; Pearson coefficients are provided in the Supplementary Materials for completeness.

## 5. Conclusions

This study demonstrates that the reproductive response of sugar beet to vernalization under speed breeding conditions follows a threshold-like pattern, with 14–15 weeks of cold exposure sharply accelerating bolting and flowering compared with 12–13 weeks. Genotypic variation was the overriding factor controlling seed yield and its components, with MARGARITA KWS and SMART DILARTA KWS identified as the most productive genotypes. In a separate mini-steckling architecture experiment, slow-release Osmocote fertilizer markedly promoted mini-steckling fresh weight, length and width, and genotype × nutrition interactions were significant for mini-steckling fresh weight and width, indicating that nutritional management can modulate the expression of genotypic differences in the root system. Multivariate analyses (PCA, CVA, Mahalanobis distances) provided quantitative support for these findings, showing clear separation of treatments and genotypes and confirming the dominant role of nutrition over genotype for mini-steckling root traits. Taken together, the findings support a strategy in which vernalization duration is matched to the genetic material and breeding objective, and nutrition is consciously managed to shape the mini-steckling phenotype. The integration of genotype-specific vernalization requirements with targeted nutrition offers a practical route toward more efficient and predictable speed breeding pipelines in sugar beet.

## Figures and Tables

**Table 1:** Effect of vernalization duration on bolting and flowering rates (stratified Cox model).

Duration (Weeks)	Bolting HR (95% CI)	Bolting *p*-Value	Flowering HR (95% CI)	Flowering *p*-Value
12	1.00 (ref *)	-	1.00 (ref)	-
13	1.51 (0.92–2.47)	0.102	1.50 (0.85–2.65)	0.158
14	3.08 (1.92–4.94)	<0.001	2.19 (1.27–3.79)	0.005
15	3.08 (1.89–5.00)	<0.001	2.66 (1.53–4.64)	<0.001

* Reference level (12 weeks of vernalization).

**Table 2:** Restricted mean survival time (τ = 100 days) to bolting and flowering.

Duration (Weeks)	Bolting RMST (Days) ± SE	Flowering RMST (Days) ± SE
12	74.3 ± 3.90	89.1 ± 2.08
13	66.4 ± 4.29	85.5 ± 2.43
14	53.4 ± 4.47	81.2 ± 2.67
15	56.3 ± 4.54	80.3 ± 2.75

**Table 3:** Selected genotype effects on bolting rate (Cox model with vernalization duration as covariate).

Genotype	Bolting HR (95% CI)	*p*-Value
0K061	1.00 (ref *)	-
MARGARITA KWS	2.80 (1.68–4.67)	<0.001
1K139	0.29 (0.14–0.57)	<0.001
SMART LIENNA KWS	0.11 (0.04–0.27)	<0.001

* Reference level (12 weeks of vernalization).

**Table 4:** Total days from sowing to bolting and flowering (estimated marginal means ± SE).

Duration (Weeks)	Days to Bolting ± SE	Days to Flowering ± SE
12	112.8 ± 1.34	149.0 ± 2.61
13	114.9 ± 1.02	151.0 ± 2.21
14	118.3 ± 0.76	154.7 ± 1.94
15	123.9 ± 0.72	159.7 ± 1.84

**Table 5:** Estimated marginal means for seed number, total seed weight and thousand-seed weight by genotype.

Genotype	Seed Number (95% CI)	Total Seed Weight (g) (95% CI)	Thousand-Seed Weight (g) (95% CI)
0K061	236 (181–308)	2.91 (2.22–3.81)	10.73 (8.80–13.07)
DESIDERIA KWS	280 (210–372)	4.13 (3.09–5.53)	14.17 (11.46–17.53)
MARGARITA KWS	361 (303–430)	5.26 (4.40–6.30)	15.24 (13.37–17.37)
SMART DILARTA KWS	285 (230–353)	4.81 (3.86–6.00)	16.46 (14.01–19.33)
SMART GINEVRA KWS	134 (63–287)	1.69 (0.79–3.64)	10.98 (6.27–19.22)
SMART SEZA KWS	251 (189–332)	4.25 (3.19–5.67)	15.26 (12.37–18.82)
1K139	219 (137–352)	1.76 (1.09–2.86)	7.39 (5.20–10.51)
SMART LIENNA KWS	232 (126–428)	2.49 (1.34–4.65)	10.91 (6.92–17.19)

**Table 6:** Two-way ANOVA for fresh weight, length and width of mini-stecklings.

Source	Sum Sq	Df	F Value	*p*-Value
	Mini-steckling fresh weight			
Nutrition	692.1	2	17.40	<0.001
Genotype	639.5	10	3.21	0.00071
Nutrition × genotype	999.1	20	2.51	0.00059
Residuals	4257.1	214		
	Mini-steckling length			
Nutrition	4.05	2	3.71	0.026
Genotype	2.22	10	0.41	0.943
Nutrition × genotype	8.75	20	0.80	0.711
Residuals	116.95	214		
	Mini-steckling width			
Nutrition	76.4	2	3.87	0.022
Genotype	341.4	10	3.46	0.00031
Nutrition × genotype	384.3	20	1.95	0.011
Residuals	2110.6	214		

**Table 7:** Estimated marginal means for fresh weight, length and width of mini-stecklings by nutrition regime.

Nutrition	Estimated Marginal Mean (95% CI)
Mini-steckling fresh weight	
Control	21.8 (20.2–23.4)
Knop + KH_2_PO_4_	20.1 (19.2–20.9)
Osmocote	34.7 (33.8–35.5)
Mini-steckling length (cm)	
Control	4.81 (4.55–5.08)
Knop + KH_2_PO_4_	5.10 (4.96–5.24)
Osmocote	5.39 (5.25–5.53)
Mini-steckling width (cm)	
Control	30.7 (29.6–31.8)
Knop + KH_2_PO_4_	28.5 (27.9–29.1)
Osmocote	36.3 (35.7–36.9)

**Table 8:** Actual number of plants in the mini-steckling experiment.

Genotype	Osmocote	Control (Knop)	Knop + KH_2_PO_4_
0K061	7	2	10
SMART IBERIA KWS	10	3	10
DUBRAVKA KWS	10	3	10
DESIDERIA KWS	10	3	10
MARGARITA KWS	10	3	10
SMART DILARTA KWS	10	3	10
1K073	10	3	10
SMART GINEVRA KWS	10	3	10
SMART SEZA KWS	9	2	10
1K139	10	3	10
SMART LIENNA KWS	10	3	10

## Data Availability

The original contributions presented in this study are included in the article/Supplementary Materials. Further inquiries can be directed to the corresponding author.

## Supplementary Materials

The following supporting information can be downloaded at: https://www.mdpi.com/article/10.3390/plants15121850/s1, Figure S1: Kaplan–Meier curves for time to flowering (a) by vernalization duration; (b) for the three representative genotypes MARGARITA KWS, SMART LIENNA KWS and 1K073; Figure S2: Residual diagnostic plots for the linear two-way ANOVA model of mini-steckling fresh weight; Figure S3: Residual diagnostic plots for the linear two-way ANOVA model of mini-steckling length; Figure S4: Residual diagnostic plots for the linear two-way ANOVA model of mini-steckling width; Figure S5: Interactive 2D PCA biplots for phenology by genotype; Figure S6: Interactive 2D PCA biplots for phenology by vernalization duration; Figure S7: Interactive 3D PCA biplot for phenology; Figure S8: Interactive 2D PCA biplots for seed traits by genotype; Figure S9: Interactive 2D PCA biplots for seed traits by vernalization duration; Figure S10: Interactive 3D PCA biplot for seed traits; Figure S11: Interactive 2D PCA biplots for mini-steckling by genotype; Figure S12: Interactive 2D PCA biplots for mini-steckling by vernalization duration; Figure S13: Interactive 3D PCA biplot for mini-steckling traits; Figure S14: Interactive 2D CVA biplots for phenology by genotype; Figure S15: Interactive 2D CVA biplots for phenology by vernalization duration; Figure S16: Interactive 2D CVA biplots for mini-steckling by genotype; Figure S17: Interactive 2D CVA biplots for mini-steckling by nutrition; Figure S18: Interactive 2D CVA biplots for seed traits by genotype; Figure S19: Interactive 2D CVA biplots for seed traits by vernalization duration; Table S1: Descriptive statistics for phenological traits and shoot architecture; Table S2: Number of plants and events (bolting, flowering) by genotype; Table S3: Detailed Kaplan–Meier estimates for time to bolting (events, RMST, median survival with 95% CI); Table S4: Number and percentage of plants reaching bolting and flowering by vernalization duration; Table S5: Comparison of hazard ratios for bolting and flowering in the main Cox model with genotype 1K073 and after excluding genotype 1K073 (sensitivity analysis); Table S6: Estimated marginal means (EMMs) for days to bolting (back-transformed from log-time model) by genotype; Table S7: Estimated marginal means (EMMs) for days to flowering (back-transformed from log-time model) by genotype; Table S8: Estimated marginal means (EMMs) for plant height (cm) by genotype; Table S9: Time-varying coefficient Cox model for bolting (sensitivity to PH violation); Table S10: Type III ANOVA results for seed traits (negative binomial for seed number; Gamma for total weight and thousand-seed weight); Table S11: Interaction test (genotype × vern_duration) for total seed weight (Gamma GLM, Type III ANOVA); Table S12: Estimated marginal means (EMMs) for seed number by vernalization duration (back-transformed from the negative binomial model); Table S13: Estimated marginal means (EMMs) for total seed weight (g) by vernalization duration (back-transformed from the Gamma model with log-link); Table S14: Estimated marginal means (EMMs) for thousand-seed weight (g) by vernalization duration (back-transformed from the Gamma model with log-link); Table S15: Simple effects of nutrition within genotypes for mini-steckling fresh weight; Table S16: Simple effects of genotype within nutrition regimes for mini-steckling fresh weight; Table S17: Kruskal–Wallis tests for mini-steckling traits; Table S18: Simple effects of nutrition within genotypes for mini-steckling width; Table S19: Simple effects of genotype within nutrition regimes for mini-steckling width; Table S20: Principal component analysis (PCA) loadings and variance explained for phenology, seed and mini-steckling traits; Table S21: Principal component analysis (PCA) angles between original variable vectors for phenology, seed and mini-steckling traits; Table S22: Principal component analysis (PCA) scores for all individual plants (phenology, seed, mini-steckling); Table S23: Canonical variate analysis (CVA) loadings and variance explained for phenology, seed and mini-steckling traits; Table S24: Canonical variate analysis (CVA) scores and variance explained for phenology (by genotype and vernalization), seed (by genotype), and mini-steckling (by genotype and nutrition); Table S25: Mahalanobis distances between group centroids for phenology (vernalization duration), seed (genotype), and mini-steckling (genotype and nutrition); Table S26: MANOVA results (Pillai’s trace) for phenology, seed and mini-steckling multivariate comparisons; Table S27: Spearman correlation matrices for phenology, seed and mini-steckling traits; Table S28: Pearson correlation matrices for phenology, seed and mini-steckling traits; Table S29: Pairwise MANOVA (Pillai’s trace) for phenology (vernalization durations) and mini-steckling root traits (nutrition regimes); Table S30: Linear discriminant analysis (LDA) classification accuracy for phenology (by genotype and vernalization duration), seed (by genotype), and mini-steckling traits (by genotype and nutrition); Table S31: Spearman rank correlations between mini-steckling and seed traits across the eight common genotypes; Table S32: Pearson correlations between mini-steckling and seed traits across the eight common genotypes.
