# Supplementary material for: Genotype, Vernalization Duration and Nutrition Interactions in Sugar Beet Speed Breeding
Source: Plants (Basel). 2026 Jun 15;15(12):1850. doi: 10.3390/plants15121850 (PMC13306349; doi:10.3390/plants15121850)
Supplement: Supplementary file 1 [file plants-15-01850-s001.zip › Figure S1. Kaplan-Meier curves for time to flowering.pdf]

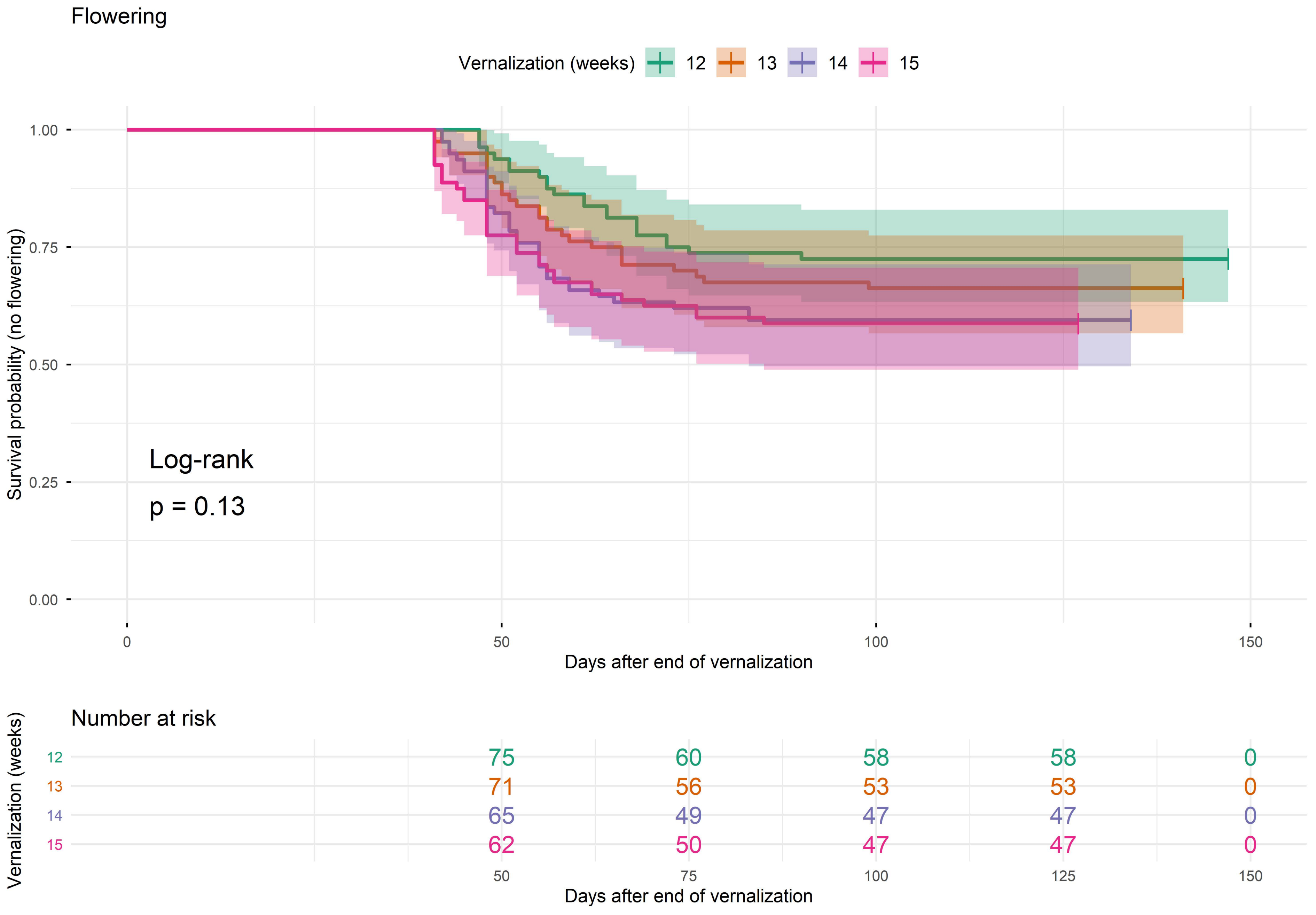
Flowering
Vernalization (weeks)
12
13
14
15
Survival probability (no flowering)
1.00
0.75
0.50
0.25
0.00
0
50
100
150
Days after end of vernalization
Log-rank
p = 0.13
Number at risk
Vernalization (weeks)
12 75 60 58 58 0
13 71 56 53 53 0
14 65 49 47 47 0
15 62 50 47 47 0
50
75
100
125
150
Days after end of vernalization

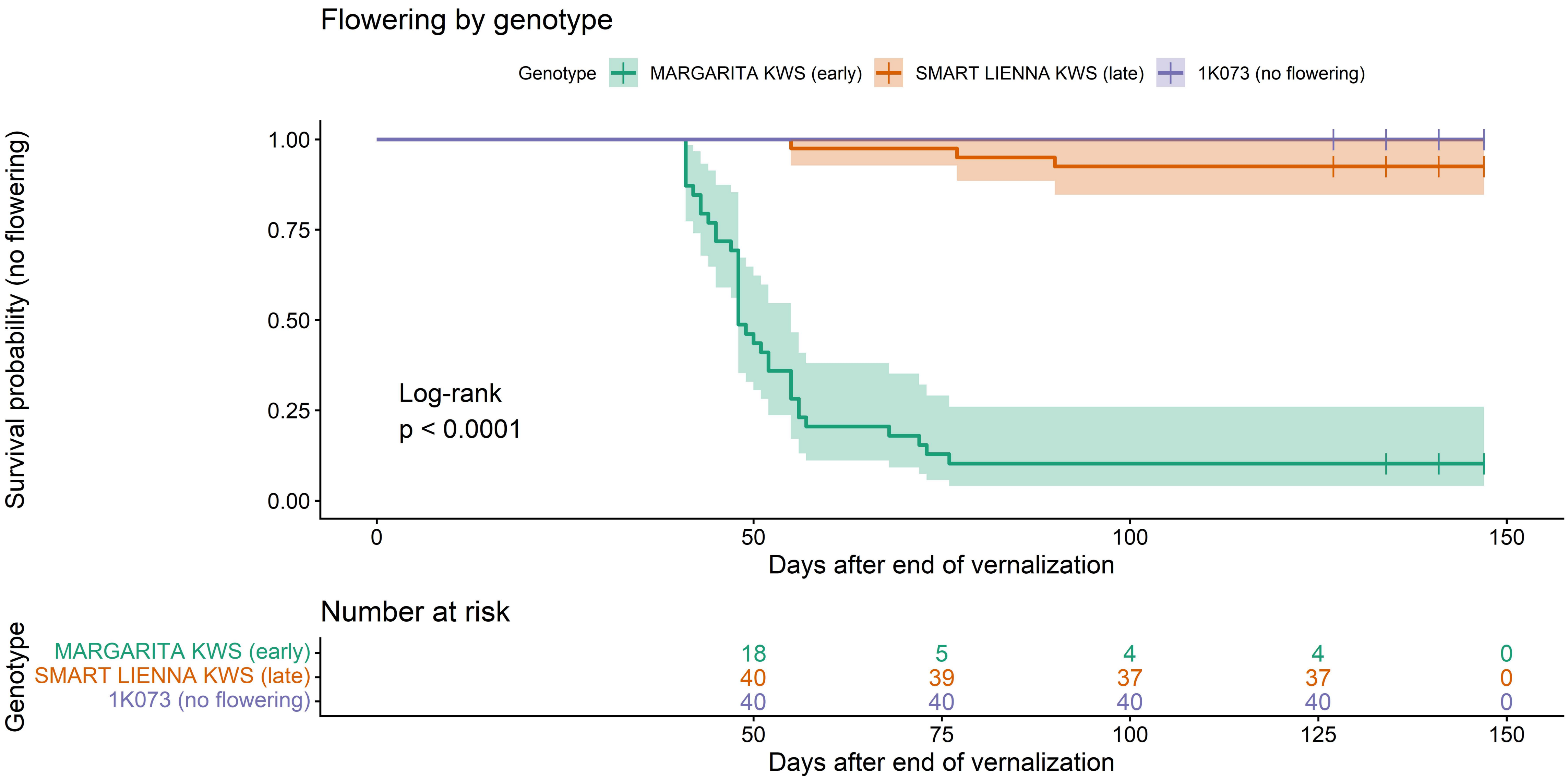

Flowering by genotype
Genotype
MARGARITA KWS (early)
SMART LIENNA KWS (late)
1K073 (no flowering)
Survival probability (no flowering)
1.00
0.75
0.50
0.25
0.00
Log-rank
p < 0.0001
0
50
100
150
Days after end of vernalization
Number at risk
Genotype
MARGARITA KWS (early)
SMART LIENNA KWS (late)
1K073 (no flowering)
18
5
4
4
0
40
39
37
37
0
40
40
40
40
0
50
75
100
125
150
Days after end of vernalization
