# Supplementary material for: Genotype, Vernalization Duration and Nutrition Interactions in Sugar Beet Speed Breeding
Source: Plants (Basel). 2026 Jun 15;15(12):1850. doi: 10.3390/plants15121850 (PMC13306349; doi:10.3390/plants15121850)
Supplement: Supplementary file 1 [file plants-15-01850-s001.zip › Figure S2. Residual diagnostic plots for the linear two‐way ANOVA model of mini‐steckling fresh weight.pdf]

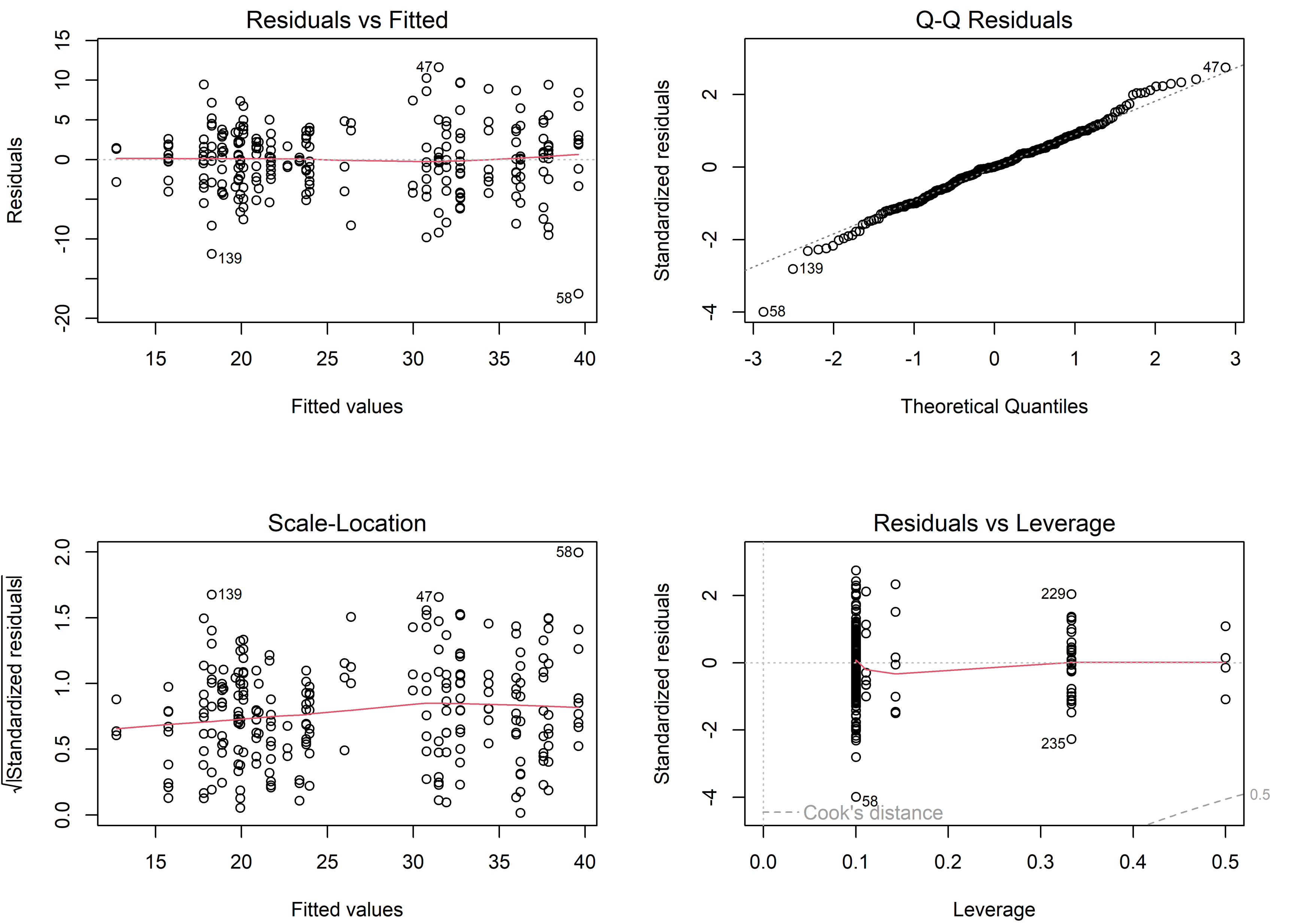
Residuals vs Fitted
Residuals
Fitted values
47
139
58
Q-Q Residuals
Standardized residuals
Theoretical Quantiles
47
139
58
Scale-Location
√|Standardized residuals|
Fitted values
58
139
47
Residuals vs Leverage
Standardized residuals
Leverage
229
235
58
Cook's distance
0.5
