# Supplementary material for: Genotype, Vernalization Duration and Nutrition Interactions in Sugar Beet Speed Breeding
Source: Plants (Basel). 2026 Jun 15;15(12):1850. doi: 10.3390/plants15121850 (PMC13306349; doi:10.3390/plants15121850)
Supplement: Supplementary file 1 [file plants-15-01850-s001.zip › Figure S3. Residual diagnostic plots for the linear two‐way ANOVA model of mini‐steckling length.pdf]

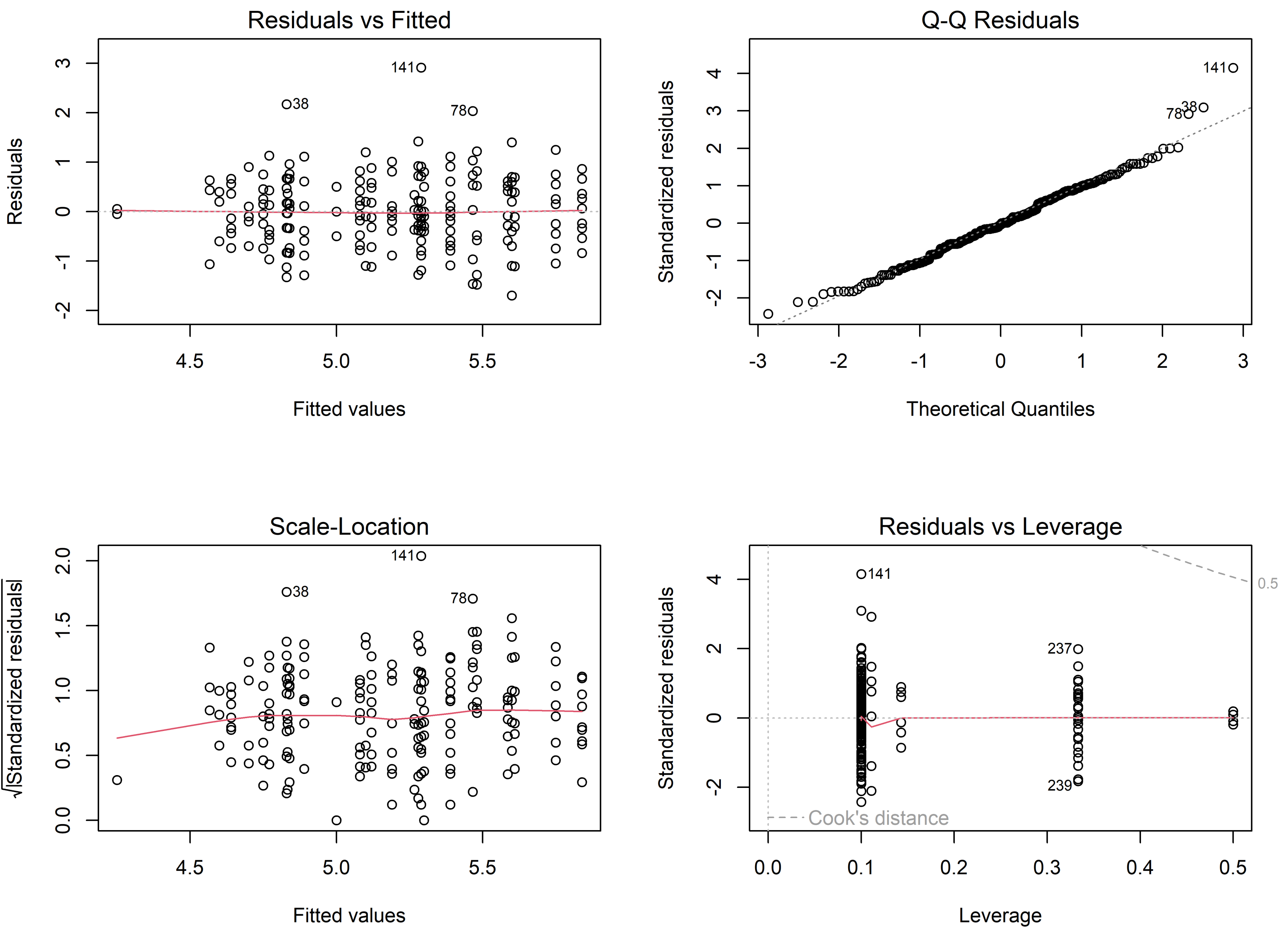
Residuals vs Fitted
Residuals
Fitted values
4.5
5.0
5.5
-2
-1
0
1
2
3
141
38
78
Q-Q Residuals
Standardized residuals
Theoretical Quantiles
-3
-2
-1
0
1
2
3
4
141
78
38
Scale-Location
√|Standardized residuals|
Fitted values
0.0
0.5
1.0
1.5
2.0
141
38
78
Residuals vs Leverage
Standardized residuals
Leverage
0.0
0.1
0.2
0.3
0.4
0.5
141
237
239
Cook's distance
0.5
