# Supplementary material for: Genotype, Vernalization Duration and Nutrition Interactions in Sugar Beet Speed Breeding
Source: Plants (Basel). 2026 Jun 15;15(12):1850. doi: 10.3390/plants15121850 (PMC13306349; doi:10.3390/plants15121850)
Supplement: Supplementary file 1 [file plants-15-01850-s001.zip › Figure S4. Residual diagnostic plots for the linear two‐way ANOVA model of mini‐steckling width.pdf]

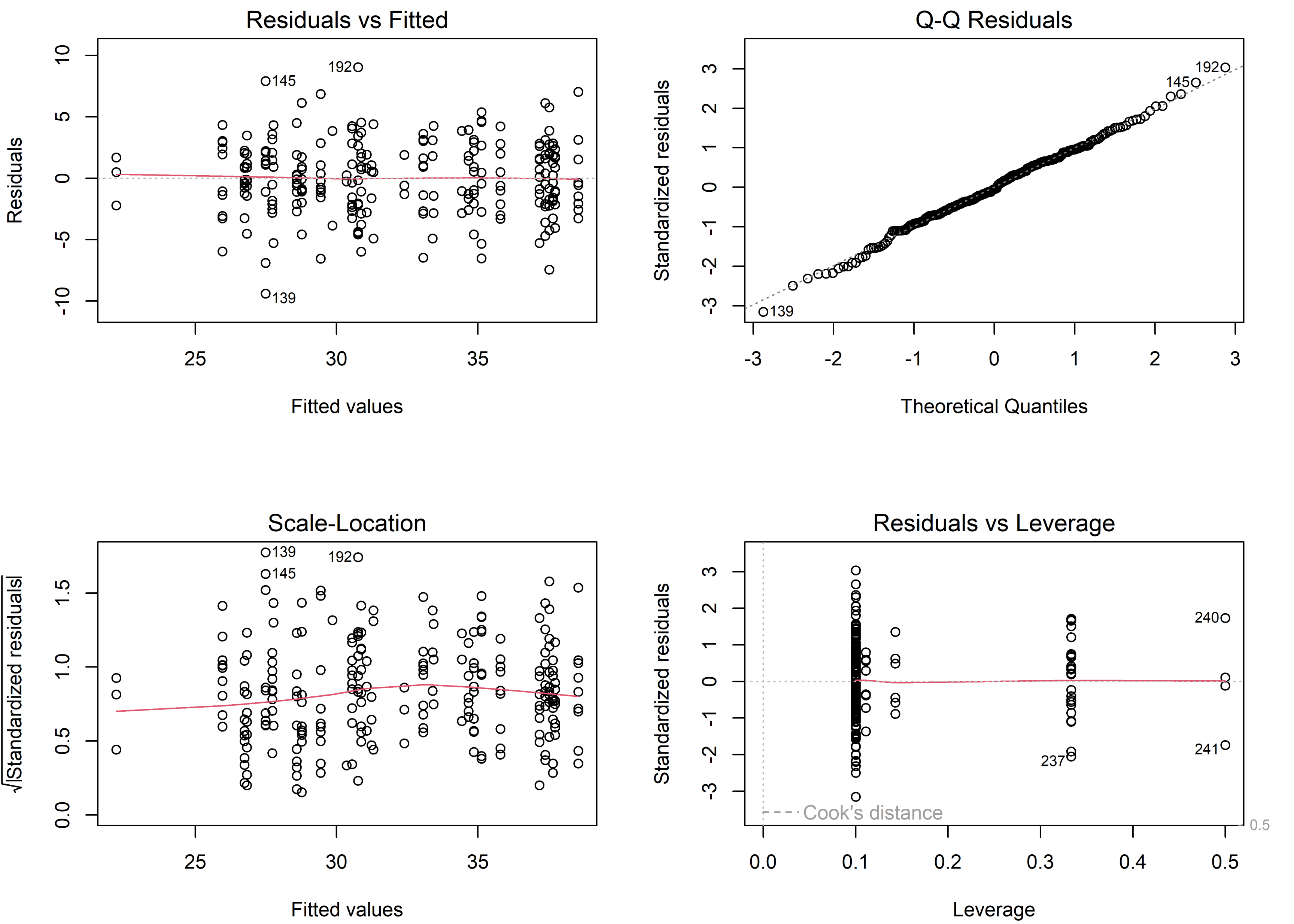
Residuals vs Fitted
Residuals
Fitted values
145
192
139
Q-Q Residuals
Standardized residuals
Theoretical Quantiles
192
145
139
Scale-Location
√|Standardized residuals|
Fitted values
139
145
192
Residuals vs Leverage
Standardized residuals
Leverage
240
241
237
Cook's distance
0.5
